# Structural and Functional Analysis of DndE Involved in DNA Phosphorothioation in the Haloalkaliphilic Archaea Natronorubrum bangense JCM10635

**DOI:** 10.1128/mbio.00716-22

**Published:** 2022-04-14

**Authors:** Wei He, Haiyan Gao, Dan Wu, Susu Jiang, Wanqiu Huang, Chao Chen, Zixin Deng, Lei Xiong, Geng Wu, Lianrong Wang

**Affiliations:** a Ministry of Education Key Laboratory of Combinatorial Biosynthesis and Drug Discovery, Hubei Clinical Center and Key Laboratory of Intestinal and Colorectal Disease, Department of Gastroenterology, Zhongnan Hospital of Wuhan University, School of Pharmaceutical Sciences, Wuhan University, Wuhan, China; b State Key Laboratory of Microbial Metabolism, School of Life Sciences and Biotechnology, Joint International Research Laboratory of Metabolic and Developmental Sciences, Shanghai Jiao Tong University, Shanghai, China; c Department of Burn and Plastic Surgery, Shenzhen Institute of Translational Medicine, Health Science Center, First Affiliated Hospital of Shenzhen University, Shenzhen Second People’s Hospital, Shenzhen, China; Oregon State University

**Keywords:** DNA phosphorothioate modification, archaeal DndE, DNA nicking, crystal structure

## Abstract

Phosphorothioate (PT) modification, a sequence-specific modification that replaces the nonbridging oxygen atom with sulfur in a DNA phosphodiester through the gene products of *dndABCDE* or *sspABCD*, is widely distributed in prokaryotes. DNA PT modification functions together with gene products encoded by *dndFGH*, *pbeABCD*, or *sspE* to form defense systems that can protect against invasion by exogenous DNA particles. While the functions of the multiple enzymes in the PT system have been elucidated, the exact role of DndE in the PT process is still obscure. Here, we solved the crystal structure of DndE from the haloalkaliphilic archaeal strain Natronorubrum bangense JCM10635 at a resolution of 2.31 Å. Unlike the tetrameric conformation of DndE in Escherichia coli B7A, DndE from N. bangense JCM10635 exists in a monomeric conformation and can catalyze the conversion of supercoiled DNA to nicked or linearized products. Moreover, DndE exhibits preferential binding affinity to nicked DNA by virtue of the R19- and K23-containing positively charged surface. This work provides insight into how DndE functions in PT modification and the potential sulfur incorporation mechanism of DNA PT modification.

## INTRODUCTION

The DNA phosphorothioate (PT) modification, a newly identified prokaryotic epigenetic mark, occurs in the DNA sugar-phosphate backbone with the nonbridging oxygen atoms being replaced by sulfur governed by DndABCDE or SspABCD machinery (1). DndABCDE proteins catalyze double-stranded DNA PTs at 4-bp consensus sequences, e.g., 5′-G_PS_AAC-3′/5′-G_PS_TTC-3′ (PS, phosphorus-sulfur linkage) in Escherichia coli B7A and Salmonella enterica serovar Cerro 87, 5′-G_PS_GCC-3′/5′-G_PS_GCC-3′ in Pseudomonas fluorescens Pf0-1, and 5′-G_PS_ATC-3′/5′-G_PS_ATC-3′ in Hahella chejuensis KCTC2396 (2–5). In contrast to these bistranded PTs, 5′-C_PS_CA-3′ in SspABCD-expressing Vibrio cyclitrophicus FF75 is a single-stranded PT modification; the complemented 5′-TGG-3′ lacks PT (6, 7). PT modification can behave as a constituent of defense systems in which sequence-specific PT is used as a recognition tag by the restriction counterparts DndFGH (8–10), PbeABCD (11), or SspE (7) to distinguish self from nonself DNA and specifically destroy non–PT-modified invading genetic parasites, resembling the role of DNA methylation in classical restriction-modification (R-M) barriers (12). In terms of R-M systems, consensus sequences in host DNA are nearly completely methylated to ensure that the self-DNA are not being attacked by the cognate restriction endonucleases (13). However, genomic mapping of PTs revealed that only 10 to 15% of the modifiable consensus motifs across prokaryotic genomes are PT protected even in the presence of active restriction counterparts, pointing to unusual PT target selection and self/nonself discrimination mechanisms (6). Notably, Dnd and Ssp systems in some bacterial strains occur in the form of orphan DndABCDE and SspABCD, respectively, which lack the restriction counterparts (3). The resulting solitary DNA PT modification has been found to have evolved additional biological functions, such as the maintenance of cellular redox homeostasis, environmental fitness, and epigenetic control of gene expression (3, 14).

Our previous exploration of PT systems in a set of halophilic archaeal strains revealed a DndCDEA-PbeABCD module in which the DndCDEA moiety confers DNA PT modification at the 5′-G_PS_ATC-3′/5′-G_PS_ATC-3′ motif and PbeABCD inhibits viral DNA replication within host cells (11). Interestingly, PbeABCD-mediated antiviral defense depends on the presence of the DNA PT modification in archaeal host DNA, which highlights a mode of action that is different from methylation-based R-M defense systems (15). It is also in sharp contrast to the defensive action of PT-based DndFGH according to the observation that PT-deficient S. enterica serovar Cerro 87 undergoes DNA damage from the unrestrained restriction activity of DndFGH, which causes growth defects and triggers the cellular SOS response (9, 10).

DndA, DndC, DndD, and DndE, encoded by the *dndA and dndBCDE* operons, catalyze the oxygen-sulfur swap in a sequence-selective and *R*_P_ stereo-specific manner (1). DndB acts as a transcriptional repressor that senses cellular ATP levels to modulate the expression of the *dndBCDE* operon (16, 17). DndA acts as a pyridoxal 5′-phosphate (PLP)-dependent cysteine desulfurase that catalyzes the conversion of l-cysteine to l-alanine and sulfane sulfur (18, 19). DndA in PT formation can be functionally replaced by an IscS orthologue, consistent with the observation that the *dndA* gene can locate either adjacent to the *dndBCDE* operon or elsewhere in prokaryotic genomes (20). DndC shows sequence homology to phosphoadenosine phosphosulfate (PAPS) reductase and exerts ATP pyrophosphatase activity *in vitro* (21, 22). DndD exhibits ATPase activity *in vitro* and is believed to provide the energy during sulfur incorporation (23). DndE, the smallest Dnd protein, is only 117 amino acid residues in length in E. coli B7A. In comparison to the extensively characterized DndA, DndB, DndC, and DndD proteins, the role of DndE in the DNA PT modification has not been explored extensively. In this study, we determined the crystal structure of the DndE protein from the haloalkaliphilic archaea Natronorubrum bangense JCM10635 at a resolution of 2.31 Å. Our structural and biochemical studies revealed that the archaeal DndE adopts a monomer conformation and is capable of nicking DNA and preferentially binding to nicked DNA, providing insight into the sulfur incorporation mechanism in Dnd systems.

## RESULTS

### Determination of the archaeal DndE structure.

**FIG 1:**
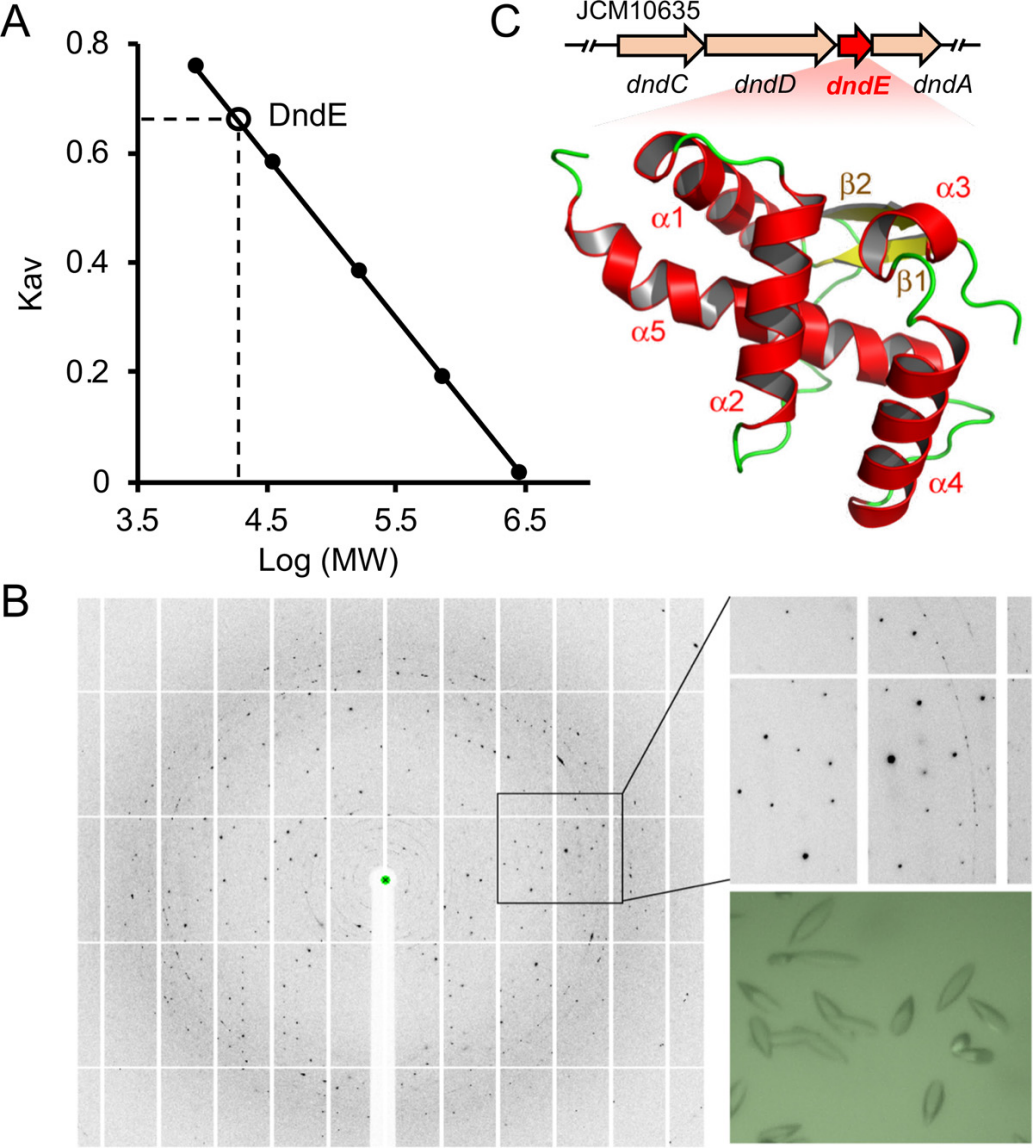
Determination of the structure of DndE from N. bangense JCM10635. (A) Size-exclusion chromatography (SEC) determination of DndE. The *x* axis represents the log value of the protein molecular weight, *K*_av_ is calculated by (*V*_e_ − *V*_o_)/(*V*_c_ − *V*_o_), *V*_e_ is the protein elution volume, *V*_c_ is the column volume, and *V*_o_ is the void volume. Superdex 200 increase 10/300 GL was used for the DndE molecular weight analysis, the column volume is 120 mL, and the void volume is 48.2 mL. The elution volume of DndE is 69 mL; thus, the molecular weight based on SEC is 16.8 kDa, which is closed to the monomer molecular weight of 15.3 kDa. (B) Crystals and diffraction image of DndE. (C) The *dndCDEA* locus and crystal structure of DndE shown in cartoon mode.

DndE can form a complex with IscS, DndC, and DndD *in vitro* to confer the DNA PT modification, and deletion of the *dndE* gene in S. enterica serovar Cerro 87 completely abolishes the PT modification at 5′-G_PS_AAC-3′/5′-G_PS_TTC-3′ (24). However, the role of DndE in DNA PT formation is unknown. To address this question, we overexpressed and purified the full-length DndE protein from a haloalkaliphilic archaeal strain N. bangense JCM10635 for crystallization and then obtained DndE crystals in the presence of 20% PEG 3350 and 0.2 M lithium acetate dihydrate (Fig. 1B); the crystal structure phase was determined by single-wavelength anomalous dispersion (SAD), and a 2.31 Å resolution was obtained with an *R*_work_/*R*_free_ factor of 18.45%/23.97% (Protein Data Bank [PDB] code 7X4E, Table S1). Size-exclusion chromatography was used for DndE aggregation state analysis (Fig. 1A), which confirmed that DndE formed a monomer consisting of the five α-helices H1 (residues 12 to 25), H2 (residues 29 to 42), H3 (residues 61 to 65), H4 (residues 69 to 83), and H5 (residues 90 to 113); two β-sheets (β1 [residues 7 to 10] and β2 [residues 56 to 60]); and a long flexible linker (residues 43 to 55) between H2 and β2 (Fig. 1C).

10.1128/mbio.00716-22.1 TABLE S1 Data collection and refinement statistics of DndE from *N. bangense* JCM10635. Download Table S1, DOCX file, 0.02 MB. Copyright © 2022 He et al. 2022 He et al. https://creativecommons.org/licenses/by/4.0/ This content is distributed under the terms of the Creative Commons Attribution 4.0 International license.

### Comparison of DndE structures from archaea and bacteria.

**FIG 2:**
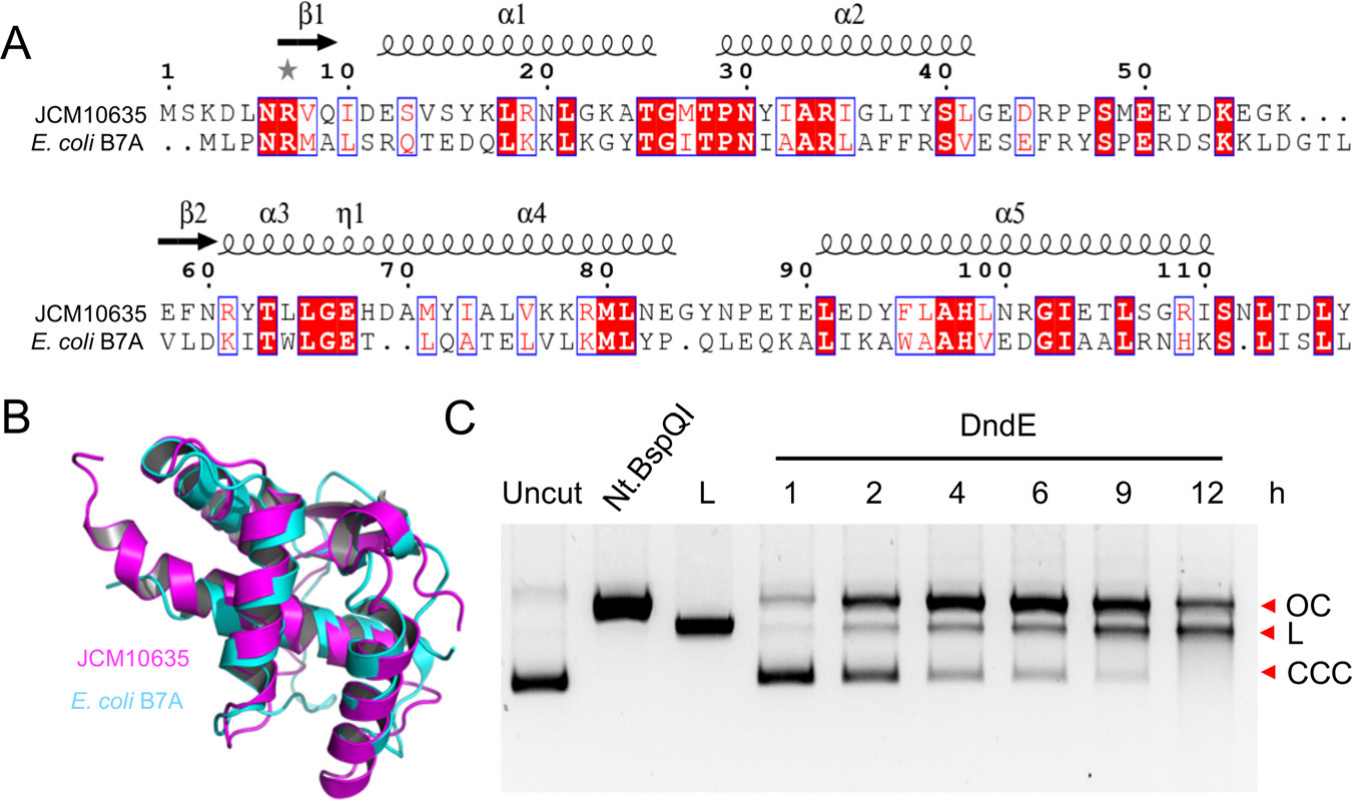
DndE crystal structure comparison and nicking activity determination. (A) Sequence alignment of DndE from N. bangense JCM10635 and E. coli B7A with the structural elements labeled on the top. (B) Structural comparison of N. bangense JCM10635 DndE and E. coli B7A DndE. DndE from N. bangense JCM10635 is in purple, and that from E. coli B7A is in cyan. (C) DNA-nicking activity of DndE; 0.3 μg of pUC19 was incubated with 6 μM DndE for 1 to 12 h, and Nt.BspQI-nicked and BamHI-linearized plasmids served as controls. OC, open circular DNA; L, linearized DNA; CCC, covalently closed circular DNA.

The crystal structure of DndE from E. coli B7A has been previously determined (PDB code 4LRV). Wild-type E. coli B7A DndE adopts a four-leaf clover-like tetrameric conformation by hydrogen bonds between the side chain of K20 in each monomer with G21 and/or G24 in the next monomer, generating a positively charged hole at the center of the tetramer, which is involved in DNA binding (25). When the positively charged region was expanded by the introduction of K21 and K24, the resultant DndE_G21/24K_ variant exhibited increased DNA binding affinity (26). Sequence alignment of DndE from bacterial E. coli B7A and archaeal N. bangense JCM10635 showed only 22% identity (Fig. 2A). However, the two DndE structures from E. coli B7A and N. bangense JCM10635 can be superimposed with a root mean square deviation of 2.138 Å over 75 Cα atoms (Fig. 2B). Unlike the tetrameric conformation of DndE from E. coli B7A, the archaeal DndE from N. bangense JCM10635 forms a monomer. The difference in the aggregation state of the archaeal DndE protein indicated Dnd protein interactions that were likely different from those in bacteria.

### DNA-nicking activity of DndE.

**FIG 3:**
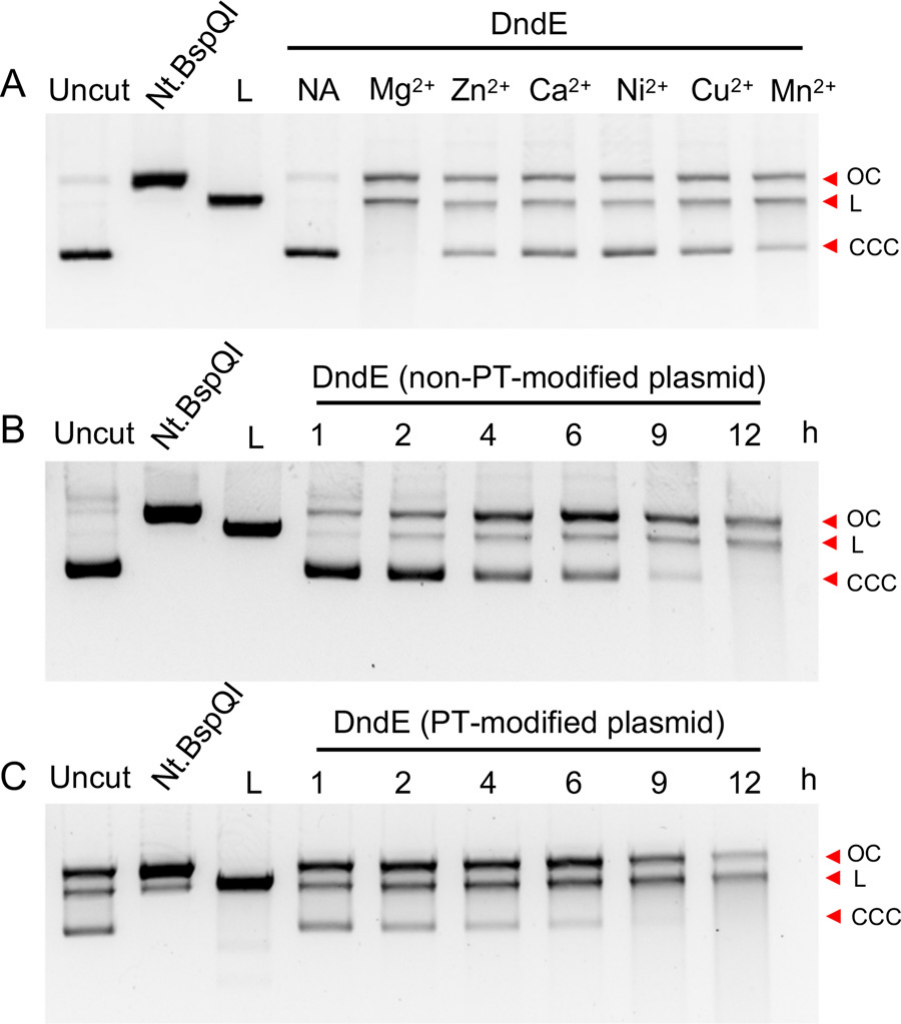
The nicking activity of DndE is dependent on divalent metal cations but is not sensitive to phosphorothioate (PT) modification. (A) pUC19 (0.3 μg) was incubated with 6 μM DndE in the reaction buffer in the presence of different divalent metal cations. Then, the reaction mixture was analyzed by 1% agarose gel. Nt.BspQI-nicked and BamHI-linearized pUC19 served as controls. NA indicates that no divalent metal cation was added. (B, C) PT-modified and non–PT-modified pUC19 (0.3 μg) were incubated with 6 μM DndE in reaction buffer. Then, the reaction mixture was analyzed by 1% agarose gel electrophoresis and stained for imaging. OC, open circular DNA; L, linearized DNA; CCC, covalently closed circular DNA.

**FIG 4:**
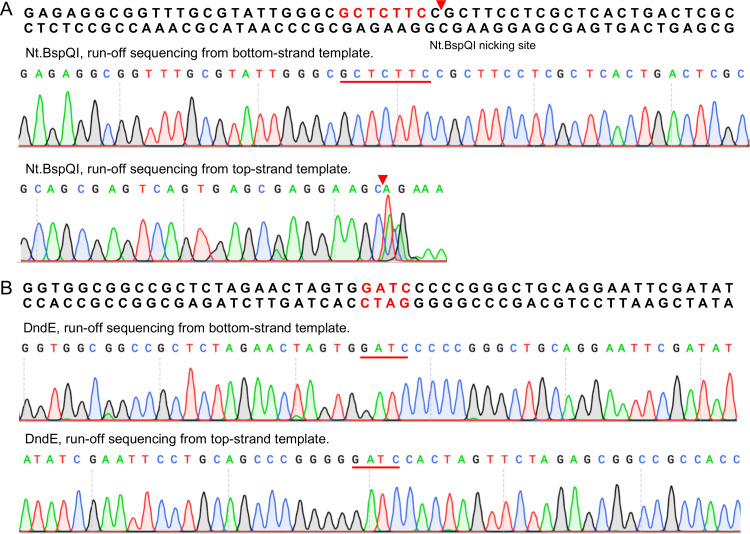
Sequence nonselectivity of DndE nicking activity. Runoff sequencing of the pUC19 plasmid nicked by Nt.BspQI (A) and DndE (B). The sequencing chromatogram disappears rapidly on the strand with the Nt.BspQI-nicked site, and the additional adenine (A) at the end of the cleavage site was added by *Taq* polymerase during sequencing. However, the sequence chromatogram map of DndE-nicked pUC19 in the presence of the 5′-GATC-3′ motif is still intact.

In SspABCD PT modification systems, SspB acts as a DNA nickase; the mutation of its DNA-nicking activity impedes PT formation (7). However, this activity has not yet been observed for Dnd proteins. Here, we were prompted to measure the nuclease activity of DndE. The *in vitro* reaction showed that DndE was capable of nicking circular pUC19 plasmid DNA to nicked products, followed by generating linearized DNA fragments (Fig. 2C). At 10 mM divalent cations, DndE was most active in the presence of Mg^2+^ and exhibited less activity with other divalent metal cations such as Mn^2+^, Ca^2+^, Zn^2+^, Ni^2+^, and Cu^2+^ (Fig. 3A). Although the PT modification occurs at 5′-G_PS_ATC-3′/5′-G_PS_ATC-3′ in N. bangense JCM10635 (11), we did not detect sequence-selective nicking at the 5′-GATC-3′ site when the gel-purified DndE-nicked pUC19 DNA was subjected to runoff sequencing (Fig. 4A and B). Together with the result that DndE had similar nicking activity against PT- and non–PT-modified pUC19 plasmids, these results demonstrate that DndE introduced nicks onto DNA in a non–sequence-specific and PT-independent manner (Fig. 3B and C). The breakage of the DNA phosphodiester bond is thus believed to be a critical step for sulfur incorporation to generate the PT modification.

### Preferentially binding to nicked DNA.

A Dali search indicated that DndE shares structural similarity to the DNA-binding proteins WYL1 (PDB code 6OAW, *Z* score = 5.5) and PARC (PDB code 1U9P, *Z* score = 5.4). WYL1 is an accessory protein in the type VI-D CRISPR-Cas system that boosts the activity of Cas13a on target RNA (27), and PARC is a transcriptional repressor in phage P22 that contains a typical helix-turn-helix (HTH) DNA-binding motif to regulate *arc* gene transcription (28). We were thus prompted to determine the interaction of DndE with fluorescently labeled DNA substrates. DndE bound to the 5′-GATC-3′/5′-GATC-3′–containing double-stranded DNA (dsDNA) with a *K_d_* = 48.4 ± 2.3 μM but exhibited no remarkable binding affinity to single-stranded DNA (ssDNA). Meanwhile, DndE exhibited comparable binding affinity to a DNA substrate that lacks the 5′-GATC-3′/5′-GATC-3′ consensus motif (dsDNA^s^; *K_d_* = 43.2 ± 2.2 μM), suggesting that DndE binds to DNA in a non–sequence-specific manner *in vitro*.

**FIG 5:**
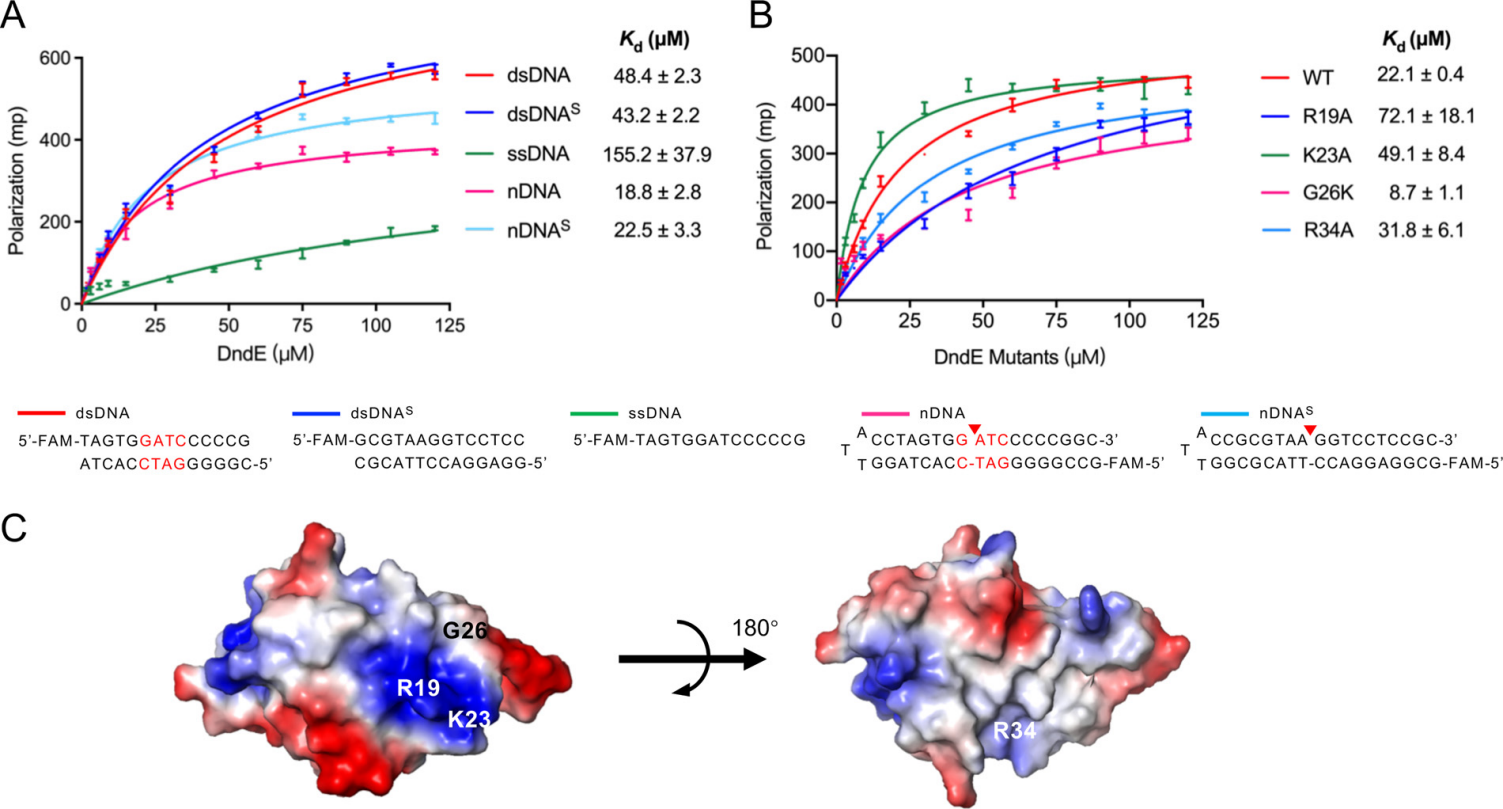
Nicked-DNA binding ability of DndE. (A) Fluorescence polarization assay for the interaction of wild-type DndE with DNA. (B) Fluorescence polarization assay for the interactions of DndE mutants with nicked DNA. (C) Electrostatic surface potential of the DndE monomer viewed in two orientations. dsDNA, double-stranded DNA; dsDNA^S^, shuffled double-stranded DNA; ssDNA, single-stranded DNA; nDNA, nicked DNA; nDNA^S^, shuffled nicked DNA.

The tetrameric DndE from E. coli B7A was previously shown to preferentially bind to nicked DNA by virtue of the positively charged lysine residues on the surface (25). Given the nicking activity of DndE *in vitro*, we also set out to assess the binding ability of archaeal DndE to DNA bearing nicks. Our results showed that DndE displayed a binding affinity of 48.4 ± 2.3 μM to intact dsDNA and showed a binding affinity of 18.8 ± 2.8 μM to nicked DNA (nDNA), which shares the same DNA sequence as dsDNA but has a nick between dG and dA within the 5′-GATC-3′/5′-GATC-3′ motif. Notably, increased binding affinity of DndE toward nicked DNA (nDNA^s^) was also detected even when the nick did not occur in 5′-GATC-3′/5′-GATC-3′. These results demonstrated that the archaeal DndE from JCM10635 behaved similarly to bacterial DndE, showing binding preference to nicked double-stranded DNA in a non–sequence-specific manner (Fig. 5A). Moreover, individual replacement of R19, K23, and R34, located in the positively charged surface, with an alanine resulted in 1.4-, 2.2-, and 3.3-fold decreases in binding affinity for nDNA, respectively. In sharp contrast, the DndE_G26K_ variant with the glycine in the positively charged surface mutated to lysine exhibited a *K_d_* value of 8.7 ± 1.1 μM, confirming that DndE_G26K_ bound 2.3-fold more strongly than wild-type DndE for nDNA (Fig. 5B). Collectively, these results revealed that DndE acts as a DNA-nicking nuclease and preferentially binds nicked DNA mediated by a positively charged patch in the surface, which provides insight into the biochemical pathway of PT generation (Fig. 5C).

## DISCUSSION

The crystal structure of DndE from E. coli B7A was a square-shaped tetramer, in which the K20 residue of each monomer extended to the center of the tetramer to produce a positively charged central hole and formed hydrogen bonds with the G21 and/or G24 residue in the next monomer to stabilize the tetramer structure (25). In contrast, the crystal structure of DndE from the haloalkaliphilic archaea N. bangense JCM10635 exhibited the conformation of a monomer rather than a tetramer, although it showed a highly similar tertiary structure to the bacterial DndE with a root mean square deviation (RMSD) value of 2.138 Å. In E. coli B7A DndE, six positively charged lysine residues are involved in binding to the nicked DNA. In terms of the archaeal DndE, the preferential binding for nicked DNA is attributed to the R19- and K23-containing positively charged surface. Mutation of the G26 residue that is in close proximity to the R19- and K23-containing positively charged surface to lysine greatly enhanced the binding affinity of DndE_G26K_ to the nicked DNA substrate because the G26K mutation enlarged the area of the positively charged surface.

There are many types of sulfur-containing nucleotides in tRNA molecules, such as 2-thiouridine (s^2^U), 2-thiocytidine (s^2^C), and 4-thiouridine (s^4^U), etc., and all tRNA thiolations that have been elucidated occur on the nucleobase moieties (21, 29). Based on whether it requires the participation of iron-sulfur clusters, tRNA thiolation can be divided into two categories: iron-sulfur cluster–dependent and iron-sulfur cluster–independent (30). For example, s^2^U34 at position 34 and s^4^U8 at position 8 are generated independently of iron-sulfur cluster formation in TusABCDE and Thil, which are responsible for the two modifications, respectively. TusABCDE and ThiI cysteine residues accept sulfur from cysteine desulfurase and transfer active sulfur directly to tRNA (31, 32). While the s^2^C modification by TtcA is an iron-sulfur cluster-dependent tRNA sulfur modification, in which IscS is responsible for providing a sulfur source, IscU is responsible for providing iron-sulfur clusters to TtcA. After accepting sulfur, TtcA transfers sulfur to generate s^2^C32 mediated by its iron-sulfur cluster (33). In comparison to tRNA thiolation, DNA PT modification is more complicated because it involves four proteins to catalyze sequence-specific sulfur incorporation. Predicted to catalyze the initial step in PT, DndA mobilizes sulfur from l-cysteine, forms an activated persulfide, and then transfers the sulfur to the Fe-S cluster of DndC. In PT-based Ssp systems, SspA and SspD exhibit cysteine desulfurase and ATP pyrophosphatase activities, respectively, resembling the functions of DndA and DndC. This raises the possibility that Dnd and Ssp systems share the same initial sulfur mobilization step but have divergent steps for DNA-target selection. The essential role of SspB’s nicking activity in the single-stranded 5′-C_PS_CA-3 modification raises a question about whether the double-stranded DNA PT formation also requires the breakage of phosphodiester bonds.

**FIG 6:**
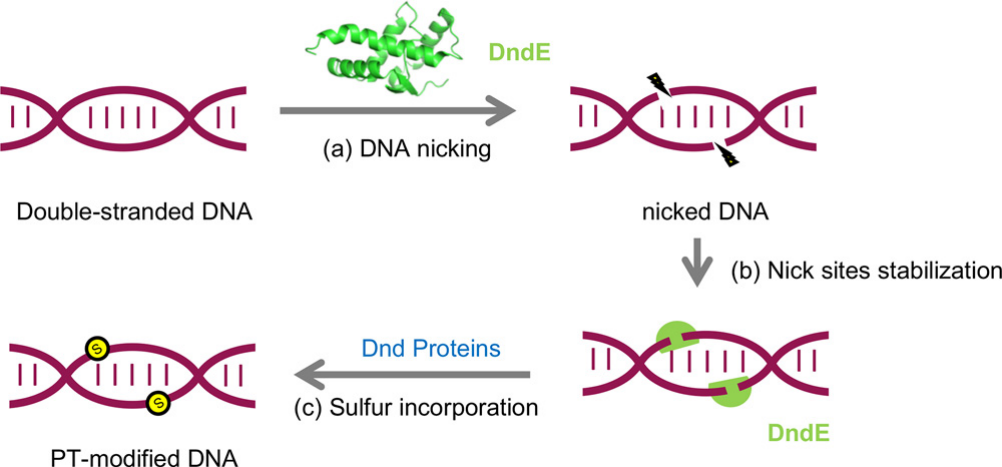
Proposed role of DndE involved in PT modification. In the proposed PT modification pathway, nicked double-stranded DNA is first generated and stabilized by DndE (a, b). Then, other Dnd proteins act on cysteine to produce and transfer sulfur into DNA to achieve stereo- and sequence-specific PT modification (c).

Indeed, our work revealed that DndE exerts nicking nuclease activity and shows preferential binding affinity to nicked DNA via a positively charged surface. Considering that DndA, DndC, DndD, and DndE form a complex to catalyze sequence-specific sulfur incorporation and DndE nicks DNA in a non–sequence-selective manner, we believe that the sequence specificity of DndE is attributed to its interaction with other Dnd proteins (Fig. 6). Collectively, this study extends our understanding about the sulfur incorporation mechanism of the DNA PT modification.

## MATERIALS AND METHODS

### Bacterial strains and plasmids.

All the bacterial strains and plasmids used in this study are listed in Table S2 in the supplemental materials.

10.1128/mbio.00716-22.2 TABLE S2 Strains and plasmids used in this study. Download Table S2, DOCX file, 0.03 MB. Copyright © 2022 He et al. 2022 He et al. https://creativecommons.org/licenses/by/4.0/ This content is distributed under the terms of the Creative Commons Attribution 4.0 International license.

### Construction of plasmids for protein purification.

A 405-bp fragment of DndE was amplified from genomic DNA of N. bangense JCM10635 by the primers DndE-10635-F/DndE-10635-R (Table S3). In addition, pET28a was digested by the NdeI and HindIII restriction enzymes, and the DndE fragment was ligated with linearized pET28a by an *in vitro* assembly method to generate pWHU3945 for wild-type DndE purification. Site-directed mutants of DndE were constructed in pWHU3945 by introducing the mutation sites in the primers, and DNA fragments with mutation sites were obtained by overlap PCR and then ligated into pET28a for protein purification.

10.1128/mbio.00716-22.3 TABLE S3 DNA oligos used in this study. Download Table S3, DOCX file, 0.02 MB. Copyright © 2022 He et al. 2022 He et al. https://creativecommons.org/licenses/by/4.0/ This content is distributed under the terms of the Creative Commons Attribution 4.0 International license.

### Construction of pWHU3940 and PT-modified pUC19.

A 5,563-bp DNA fragment harboring *dndBCDE* from H. chejuensis KCTC2396 was amplified by the primers 2396-BCDE-F/2396-BCDE-R (Table S3) from genomic DNA of H. chejuensis KCTC2396. Moreover, pACYC184 was digested with BamHI and SalI, and the *dndBCDE* fragment was ligated into pACYC184 by an *in vitro* assembly method to generate pWHU3940. pUC19 and pWHU3940 were cotransformed into E. coli DH10B to generate PT-modified pUC19. Then, the two-plasmid mixture was extracted, and PT-modified pUC19 was separated and purified by agarose gel electrophoresis.

### Protein expression and purification.

The DNA fragment with the full-length *dndE* gene from N. bangense JCM10635 was subcloned into the pET28a vector with an N-terminal His tag, and the resulting plasmid was transformed into E. coli BL21(DE3) cells, which were then cultured at 37°C to an optical density at 600 nm (OD_600_) of 0.6 to 0.8. The cell culture was cooled at 4°C, and then 0.2 mM isopropyl β-d-1-thiogalactopyranoside (IPTG) was added, and the cells were incubated at 16°C for 18 h for protein expression. The cells were collected and resuspended in wash buffer (25 mM Tris-HCl [pH 8.0], 150 mM NaCl, 20 mM imidazole) and then disrupted by a homogenizer (JNBIO, Guangzhou, China). The cell lysate was centrifuged at 16,000 × *g* for 1 h at 4°C to remove cell debris. After centrifugation, the supernatant was loaded on Ni^2+^-nitrilotriacetic acid (Ni^2+^-NTA) affinity beads (Yeasen, Shanghai, China), washed with wash buffer, and eluted with elution buffer (25 mM Tris-HCl [pH 8.0], 150 mM NaCl, 200 mM imidazole). Then, the eluted protein was purified by size-exclusion chromatography with a Superdex 200 gel filtration column (GE Healthcare, Uppsala, Sweden) in lysis buffer (25 mM Tris-HCl [pH 8.0], 150 mM NaCl, 2 mM DTT). Finally, the peak fractions were collected and concentrated at 10 mg/mL for crystallization.

### Crystallization, data collection, and structure determination.

N. bangense JCM10635 DndE crystals were grown by the hanging-drop vapor-diffusion method with buffer containing 20% PEG 3350 and 0.2 M lithium acetate dihydrate (Hampton Research, USA) at 14°C. The crystals were stored in liquid nitrogen with cryoprotectant buffer containing 25% glycerol. Crystal diffraction data at a resolution of 2.3 Å were collected on the BL19U1 Beamline at the National Center for Protein Sciences Shanghai (NCPSS, Shanghai, China) at 100 K and processed by HKL3000 software. The PHASER program was used to determine the DndE crystal structure by the SAD method. Model building and refinement were performed using COOT and REFMAC. The crystals belonged to the P6422 space group, and there was one molecule of DndE in each asymmetric unit. The final refined model had an *R*_work_/*R*_free_ of 18.45%/23.97%. The quality of the structure model was evaluated by the PROCHECK program, and the results indicated that the model exhibited good stereochemistry based on a Ramachandran plot.

### DNA-nicking assays.

DNA-nicking assays were carried out with 0.3 μg of plasmid DNA in 100 mM NaCl, 50 mM Tris-HCl (pH 8.0), 10 mM MgCl_2_, or other divalent metal cations (MnCl_2_, NiSO_4_, CaCl_2_, ZnCl_2_, CuSO_4_), 0.1 mg bovine serum albumin (BSA) and 6 μM DndE in a total volume of 20 μL. The reactions were performed at 37°C for 2 to 12 h and quenched by adding 2 μL of 10× gel loading dye (Yeasen, Shanghai, China), followed by 1% DNA agarose gel electrophoresis.

### Runoff sequencing.

Runoff sequencing was used to verify the DNA cleavage site. The DndE and Nt.BspQI digestion products were extracted and purified for DNA sequencing. A pair of primers, RO_F/RO-R (Table S3), was used for double-stranded sequencing; Nt.BspQI, a nickase, was used as a control; and the cleavage site was located on 5′-GCTCTTCN↓-3′. The sequencing chromatogram was weak near the nick site, and an A tail was added by *Taq* polymerase.

### DNA nick site binding assay.

A fluorescence polarization (FP) method was used for determination of the binding affinity of DndE for substrate DNA. FP reactions were carried out with 10 nM fluorescein-labeled DNA substrate and serial dilutions of DndE in reaction buffer (100 mM NaCl, 50 mM Tris-HCl [pH 8.0]) at room temperature in a total volume of 200 μL and then measured with an excitation wavelength of 490 nm and an emission wavelength of 535 nm by using the Biotek Synergy H1 platform (Agilent, CA, USA). The dissociation constant (*K_d_*) was analyzed by Prism 9 by nonlinear least-squares analysis.

### Data availability.

The atomic coordinates and structural factors of DndE from N. bangense JCM10635 have been deposited in the Protein Data Bank under the accession number 7X4E.
