# Supplementary material for: Structural and Functional Analysis of DndE Involved in DNA Phosphorothioation in the Haloalkaliphilic Archaea Natronorubrum bangense JCM10635
Source: mBio. 2022 Apr 14;13(3):e00716-22. doi: 10.1128/mbio.00716-22 (PMC9239217; doi:10.1128/mbio.00716-22)
Supplement: TABLE S1 [file mbio.00716-22-s0001.docx]

**Supplementary Table 1.** Data collection and refinement statistics of DndE from *N. bangense* JCM10635.

|  | *N. bangense* JCM10635-DndE |
| --- | --- |
| **Data collection** |  |
| Space group | P6422 |
| Unit cell dimensions |  |
| *a*, *b*, *c* (Å) | *a*=69.732 Å, *b*=69.732 Å, *c*=106.022 Å |
| α, β, γ (°) | α=β=90°, γ=120° |
| Wavelength | 0.97778 Å |
| Resolution (Å) | 50.00-2.31 (2.39-2.31) |
| *R*_merge_ | 0.458 (0.07931) |
| *CC1/2* | 0.760 |
| *I/σ_I_* | 24.8 (1.07) |
| Completeness (%) | 100.0 (100.0) |
| Redundancy | 34.7 (29.0) |
| **Refinement** |  |
| Resolution (Å) | 60.39-2.32 |
| Number of reflections | 7040 |
| *R*_work_/*R*_free_ | 18.45%/23.97% |
| Number of atoms | 925 |
| Number of protein atoms | 902 |
| Number of water atoms | 23 |
| *B*-factors (overall) | 37.922 |
| *B*-factors (protein) | 36.427 |
| *B*-factors (water) | 96.561 |
| RMSD bond lengths (Å) | 0.0104 |
| RMSD bond angles (°) | 1.4324 |
| Ramachandran plot statistics |  |
| Most favorable | 99.1% |
| Additionally allowed | 0.9% |
| Disallowed | 0% |

R_merge_ = Σ_h_Σ_i_ |*I*_h,i_ – *I*_h_|/Σ_h_Σ_i_ *I*_h,i_ for the intensity (*I*) of observation i of reflection h. R factor = Σ||*F*_obs_| - |*F*_calc_||/Σ|*F*_obs_|, where *F*_obs_ and *F*_calc_ are the observed and calculated structure factors, respectively. R_free_ = R factor calculated using 5% of the reflection data chosen randomly and omitted from the start of refinement. RMSD = root-mean-square deviation from ideal geometry. Data for the highest resolution shell are shown in parentheses.
