# Supplementary material for: Structural and Functional Analysis of DndE Involved in DNA Phosphorothioation in the Haloalkaliphilic Archaea Natronorubrum bangense JCM10635
Source: mBio. 2022 Apr 14;13(3):e00716-22. doi: 10.1128/mbio.00716-22 (PMC9239217; doi:10.1128/mbio.00716-22)
Supplement: TABLE S2 [file mbio.00716-22-s0002.docx]

**Supplementary Table 2. Strains and plasmids used in this study**

| **Name** | **Characteristics** | **Source or reference** |
| --- | --- | --- |
| **Strains** |  |  |
| *N. bangense* JCM10635 | d(G_PS_A), GeneBank: NZ_CP031305.1 | (1) |
| *E. coli* BL21(DE3) | *F- ompT gal dcm lon hsdSB*(*rB-mB-*) λ(DE3 [*lacI* *lacUV5*-T7p07 *ind*1 *sam*7 *nin*5]) [malB^+^]K-12(λ^S^) | Novagen |
| *E. coli* DH10B | *F^-^ endA*1 *recA*1 *galE*15 *galK*16 *nupG* *rpsL* ΔlacX74 Φ80lacZΔM15 *araD*139 *Δ(ara, leu)*7697 *mcrA* Δ(*mrr- hsdRMS-mcrBC*) λ^-^ | Novagen |
| **Plasmids** |  |  |
| pWHU3940 | Derivative of pACYC184, expressing *dndBCDE* from *H. chejuensis* KCTC2396 | This work |
| pWHU3945 | pET28a derivative, expressing wild-type DndE from *N. bangense* JCM10635 | This work |
| pWHU3946 | pET28a derivative, expressing DndE_R19A_ from *N. bangense* JCM10635 | This work |
| pWHU3949 | pET28a derivative, expressing DndE_K23A_ from *N. bangense* JCM10635 | This work |
| pWHU3950 | pET28a derivative, expressing DndE_G26K_ from *N. bangense* JCM10635 | This work |
| pWHU3952 | pET28a derivative, expressing DndE_R34A_ from *N. bangense* JCM10635 | This work |

**REFERENCE**

1. Xiong L, Liu S, Chen S, Xiao Y, Zhu B, Gao Y, Zhang Y, Chen B, Luo J, Deng Z, Chen X, Wang L, Chen S. 2019. A new type of DNA phosphorothioation-based antiviral system in archaea. Nat Commun 10:1688.
