# Supplementary material for: Structural and Functional Analysis of DndE Involved in DNA Phosphorothioation in the Haloalkaliphilic Archaea Natronorubrum bangense JCM10635
Source: mBio. 2022 Apr 14;13(3):e00716-22. doi: 10.1128/mbio.00716-22 (PMC9239217; doi:10.1128/mbio.00716-22)
Supplement: TABLE S3 [file mbio.00716-22-s0003.docx]

**Supplementary Table 3. DNA oligos used in this study**

| **Primers** | **Sequences** |
| --- | --- |
| DndE-10635-F | GTGCCGCGCGGCAGCCATATGAGTAAAGACCTCAAC |
| DndE-10635-R | GAGTGCGGCCGCAAGCTTTCATGATTCCGTCGCAGA |
| 2396-BCDE-F | CACCCGTCCTGTGGATCCAGCCATCACCACTCGTAA |
| 2396-BCDE-R | TCAAGGGCATCGGTCGACTGATCTGACAGTAACCTC |
| RO_F | TCCTGGCCTTTTGCTGGC |
| RO_R | CTTCGCTATTACGCCAGC |
| **Oligos for nicked-DNA binding assay** | |
| E-D1-F | 5’-FAM-TAGTGGATCCCCCG |
| E-D1-R | CGGGGGATCCACTA |
| E-D2-F | 5’-FAM-GCGTAAGGTCCTCC |
| E-D2-R | GGAGGACCTTACGC |
| E-D3-F | 5’-FAM-GCCGGGGGATCCACTAGGTTACCTAGTGG |
| E-D3-R | ATCCCCCGGC |
| E-D4-F | 5’-FAM-GCGGAGGACCTTACGCGGTTACCGCGTAA |
| E-D4-R | GGTCCTCCGC |
